# GFP Facilitates Native Purification of Recombinant Perlucin Derivatives and Delays the Precipitation of Calcium Carbonate

**DOI:** 10.1371/journal.pone.0046653

**Published:** 2012-10-03

**Authors:** Eva Weber, Christina Guth, Ingrid M. Weiss

**Affiliations:** INM – Leibniz Institute for New Materials gGmbH, Saarbruecken, Germany; Griffith University, Australia

## Abstract

Insolubility is one of the possible functions of proteins involved in biomineralization, which often limits their native purification. This becomes a major problem especially when recombinant expression systems are required to obtain larger amounts. For example, the mollusc shell provides a rich source of unconventional proteins, which can interfere in manifold ways with different mineral phases and interfaces. Therefore, the relevance of such proteins for biotechnological processes is still in its infancy. Here we report a simple and reproducible purification procedure for a GFP-tagged lectin involved in biomineralization, originally isolated from mother-of-pearl in abalone shells. An optimization of *E. coli* host cell culture conditions was the key to obtain reasonable yields and high degrees of purity by using simple one-step affinity chromatography. We identified a dual functional role for the GFP domain when it became part of a mineralizing system *in vitro*. First, the GFP domain improved the solubility of an otherwise insoluble protein, in this case recombinant perlucin derivatives. Second, GFP inhibited calcium carbonate precipitation in a concentration dependent manner. This was demonstrated here using a simple bulk assay over a time period of 400 seconds. At concentrations of 2 µg/ml and higher, the inhibitory effect was observed predominantly for HCO_3_^−^ as the first ionic interaction partner, but not necessarily for Ca^2+^_._ The interference of GFP-tagged perlucin derivatives with the precipitation of calcium carbonate generated different types of GFP-fluorescent composite calcite crystals. GFP-tagging offers therefore a genetically tunable tool to gently modify mechanical and optical properties of synthetic biocomposite minerals.

## Introduction

Biomineralization offers a rich source of inspiration for materials science and biotechnology. Natural biominerals were adapted during the time-course of evolution over millions of years to their specific functions. In order to achieve control over mineralization, many organisms use amorphous mineral precursor phases [1], [2]. They form hierarchical composite architectures and complex morphologies by cell-biological and chemical molding [3], [4], [5], [6], [7]. The impact of the microenvironment and an organic matrix with tunable solubilities is widely studied as one of the key mechanisms to understand biomineralization [2], [8], [9], [10], [11]. Several biopolymers with specific functions in terms of mineralization are involved in these extremely complex processes. Especially proteins extracted from a wide range of natural biominerals are in the current focus of multiple research efforts. However, the amount of proteins available from native biominerals is often limited. Some of them can be extracted by using classical purification procedures, e.g. [12], [13], and molecular genetic approaches [14], [15]. However, these may not represent the most active forms in a dynamic environment. Electrostatic conditions and the ionic strength certainly change while mineral phases convert from less stable to more stable polymorphs. The molecular design of the biomolecules involved in mineralization may account for such changes. For example, the prominent hydroxyapatite inhibitor osteopontin is differentially regulated by phosphorylation in milk and bone [16]. Many other biochemical mechanisms involved in biomineralization, especially mollusc shell proteins, are by far less understood. In order to understand how exactly such unconventional proteins fulfill their functional role in biomineralization one possible strategy would be to express the genes of interest in sufficient amounts in suitable host organisms. In addition, one could mimic the processing of the target molecule under the conditions of extracellular mineralization.

The expression of biomineralization genes in biotechnologically attractive hosts such as *E. coli* requires paying attention to the fact that proteins involved in biomineralization can exist in soluble, semi-soluble and insoluble variants as part of their function. An adaptation and/or simplification of purification steps is required in order to mask and/or maintain certain surface interactions and assembly properties of the protein. Perlucins, a well-known example, represent a lectin protein family from nacre or mother-of-pearl [14], [15], [17]. The N-terminal part of the native protein (155 amino acids, 17 kDa) is glycosylated and soluble. It was originally isolated from *Haliotis laevigata* shell extracts [13]. Perlucins may vary in their C-terminus, which contains up to 9 SLHANLQQRD repeats (some amino acids exchanged, e.g. A vs. G in some motifs) in a 240 amino acid precursor protein [14]. Perlucin from *Haliotis laevigata* preferably incorporates into calcium carbonate *in vitro* and interferes with the growth pattern, but not the atomic lattice of calcite as revealed by AFM in supersaturated calcium carbonate solution in the presence of 10 µg/ml perlucin [18]. The natively from nacre (mother-of-pearl) extracted perlucin is a functional C-type lectin with an unusually broad spectrum of sugar binding: Both, mannose and galactose residues were recognized between pH 5.5–7 [17]. After cleavage of the signal peptide, perlucin contains six cysteines, which were recently suggested to interfere with calcium carbonate precipitation [19]. Perlucin seems to interact more or less specifically with different surfaces (-CH_3_, -NH_2_, -COOH, -OH terminated self-assembled monolayers, SAMs). A flexible unfolding of perlucin in the presence of -CH_3_ terminated SAMs or phospholipid monolayers was suggested according to surface plasmon resonance (SPR) measurements. It remains unclear whether or not this promotes the nucleation of CaPO_4_ seeds [20]. Recombinant perlucin was previously obtained using prokaryotic expression systems, however its purification required a technically elaborate denaturing polyacrylamide gel electrophoresis step [21]. Subsequent gentle DTT renaturing steps recover the mineralization activity of perlucin with respect to calcite nucleation [21]. Wang and colleagues achieved natively purified forms of perlucin by recombinant fusion to maltose binding protein and demonstrated that these perlucins interfere with calcite crystals [15]. The perlucin N-terminus is therefore a promising candidate to overcome inhibitory effects of other proteins on mineralization, as previously demonstrated for natively extracted nacre protein mixtures [22], [23].

In this context, we investigated the potential influence of His-tagged GFP when fused to target sequences similar to perlucin. Various crystals tend to incorporate small chemical dyes [24], [25], and also GFP incorporates into alpha-lactose monohydrate crystals *in vitro* [26], [27]. In a recent report, histidine-tagged GFP co-precipitated with a metastable vaterite phase and the fluorescent organic matrix became subsequently insoluble while the calcium carbonate dissolved. The intrinsic fluorescence of the protein was conserved during its interaction with the mineral phase, indicating a proper folding of GFP in its insoluble state [28]. This paper reports on perlucin derivatives in the form of GFP fusion proteins, obtained by heterologous expression in *E. coli*, and their interaction with calcium carbonate precipitation from solution. The incorporation of GFP into mineral precipitates was investigated using fluorescence microscopy techniques. The influence of the GFP domain on the solubility of heterologously expressed fusion proteins, and its interaction with calcium carbonate precipitation was investigated as a function of concentration and the type of the first ionic reagent.

## Results

### Expression and Characterization of Perlucin-tagged GFPs

In order to study the influence of a soluble GFP domain on mineralization, we constructed fusion proteins with a common histidine affinity tag for a fast and simple one-step affinity purification using Ni^2+^-NTA resins. The protein sequences were designed in such a way that the affinity tag was located at the N-terminus followed by a central GFP domain. Our working hypothesis was that incorporating a GFP domain would increase the solubility of recombinant proteins involved in biomineralization and thus potentially mimic the role of glycosylation, which is often observed in natural proteins involved in biomineralization. Since affinity tags such as 6xHis could also influence a potential interaction with mineral precursors, these tags were topographically separated from the biomineralization-related protein. One specific goal of this study was to take advantage of the intrinsic fluorescence of the GFP domain in order to localize them during their interaction with mineral phases while they are forming. The fluorescence would also help to identify whether and how proteins involved in biomineralization would form insoluble protein aggregates as part of a putative ripening process of the composite material.

**Figure 1:**
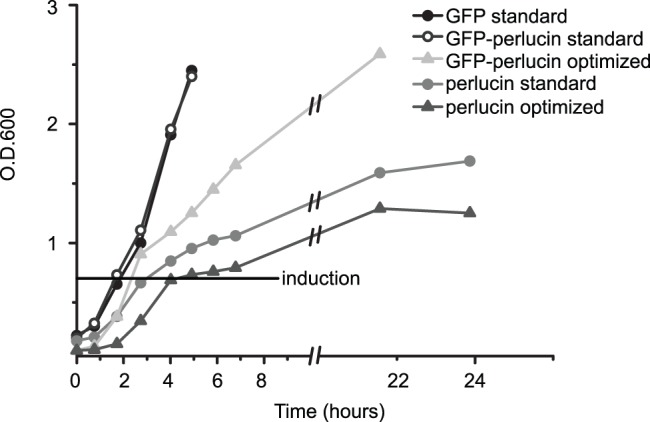
Cell density profiles for recombinant GFP, GFP-perlucin and perlucin expressing *E. coli* cultures. Standard culture conditions (circles) are suitable with respect to both, cell growth and protein yield for GFP (black line, closed circles). Despite the fast growth rate of GFP-perlucin cells under standard conditions (black line, open circles), the final protein yield is low (Figure 2B). When these cells are cultivated using the optimized temperature profile, cell densities reach an O.D. (optical density) of ∼2.6 after 22 hours (light grey triangles) and protein yields increase (Figure 2C). Perlucin expressing cells divide slower under both, standard (grey circles) and optimized (dark grey triangles) growth conditions to a maximum O.D. of 1.6 after 24 hours, suggesting a possible toxic effect of perlucin on cell proliferation. Regardless of culture conditions, recombinant perlucin can hardly be purified natively (Supporting Information file Figure S1).

The prokaryotic expression vectors used in this study contained open reading frames with verified sequences for His-tagged perlucin, His-tagged GFP (GFP), and His-tagged GFP-perlucin (GFP-perlucin). The latter two were in the main focus of this study. The expression of His-tagged perlucin without GFP in *E. coli* leads to a reduced growth rate and to a final O.D. of 1.8 (Figure 1 and Figure S1). Although protein expression slightly increased under optimized conditions (Figure S1, bottom right), a native purification test showed that the protein is not soluble under the culture conditions used here. Recombinant GFP-perlucin protein produced in *E. coli* under standard conditions was detected mainly as part of the insoluble fractions of cell extracts (Figure S1).

**Figure 2:**
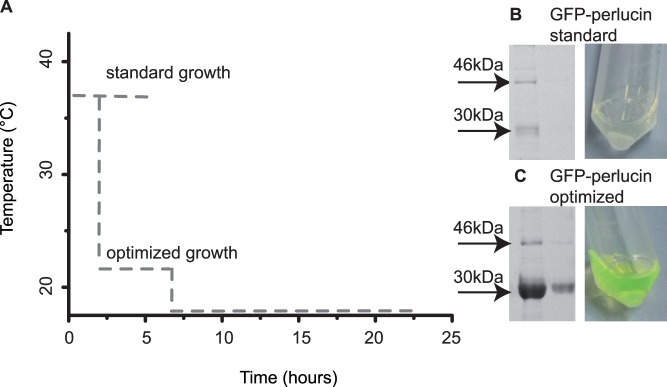
Culture conditions for native GFP-perlucin expression. (A) Temperature profile for two different growth conditions. (B) The native protein yield from cultures grown under standard conditions was low, inclusion bodies were favoured. (C) Optimized culture conditions yielded about 10× more soluble full length GFP-perlucin, (indicated by a mean value of 46 kDa) and about 100× more truncated GFP-perlucin (indicated by a mean value of 30 kDa) as quantified by coomassie gels. See for the exact molecular mass Figure 3 and for the molecular weight standard Supporting Information file Figure S1.

**Figure 3:**
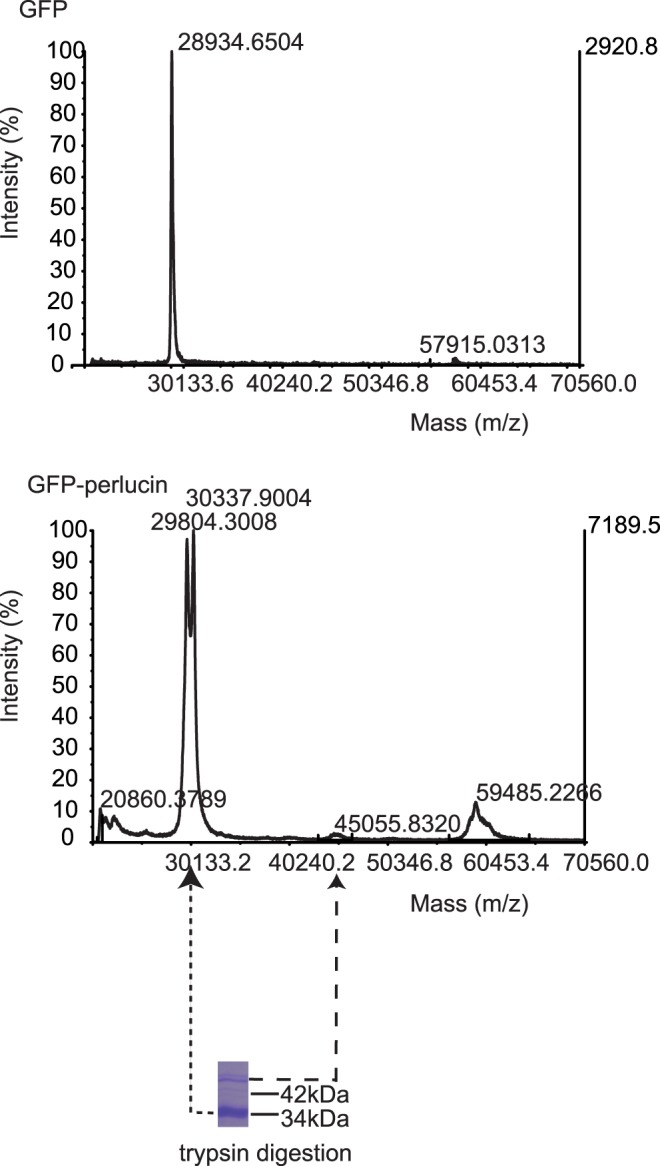
MALDI-TOF/TOF (MS+MS/MS) of GFP-perlucin variants. Intact mass analysis of Ni^2+^-NTA eluates of His-tagged GFP (top) reveals the expected molecular mass of 28.9 kDa. His-tagged GFP-perlucin (bottom) yields two major peaks at 29.8 kDa and 30.3 kDa corresponding to truncated protein variants. A minor broad peak at 45 kDa indicates the presence of the 395 amino acid protein (see also Figure 4). Protein dimers could explain additional intact mass peaks in spectra of GFP (57.9 kDa, top) and GFP-perlucin (59.4 kDa, bottom).

**Figure 4:**
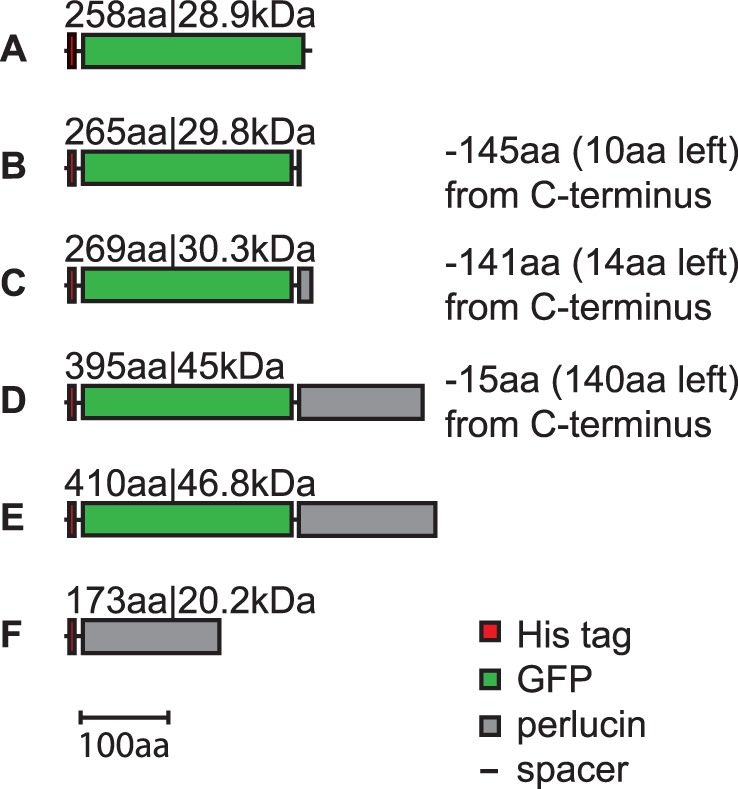
GFP, GFP-perlucin and perlucin variants achieved by protein expression and purification. The obtained GFP-perlucin derivatives as identified by MALDI-TOF/TOF (MS+MS/MS), either intact or after trypsin digestion, are His-tagged GFP (A), and truncated GFP-perlucins lacking 141 or 145 (B,C), or 15 C-terminal amino acids (D). Mass fragments of tryptic peptides extracted after SDS-PAGE identified the 46.8 kDa protein as the full length His-tagged GFP-perlucin including the two C-terminal repeat motifs (E). His-tagged perlucin without GFP domain (F) was not analyzed.

Native extraction of the protein was achieved when the culture conditions were optimized by reducing IPTG concentration for induction to 0.5 mM and adjusting temperatures to 22°C for 8 hours and 18°C for 16 hours (Figure 1 and 2, Figure S1). A one-step Ni^2+^-NTA affinity purification from 8 ml crude cell extract yielded 100 µg protein eluate, which predominantly contained a 34 kDa fragment and a minor protein band at 47 kDa according to SDS-PAGE (Figure S1, top right, lane E1). The latter corresponded to the calculated mass of 46.8 kDa (28.9 kDa for GFP and 17.9 kDa for perlucin). Mass spectrometric analyses of the trypsin digested 47 kDa band confirmed the identity of the full-length protein, including the C-terminal “DSLHANLQQR” fragment (Table 1). Intact mass analysis revealed a minor 45 kDa mass peak (Figure 3, right arrow), which presumably corresponded to a GFP-perlucin derivative lacking 15 amino acids. The 34 kDa band (Figure 3, left arrow) corresponded to several truncated protein variants of 29.8 and 30.3 kDa as confirmed by intact mass spectra. Figure 4 provides a schematic overview of all His-tagged GFP-perlucin variants as identified by mass spectroscopy.

**Table 1:** MALDI-TOF/TOF (MS+MS/MS) identification of GFP-perlucin (46.8 kDa).

Sequence	Calculated mass	Expected mass
DEDSFIR	881.3999	881.4128
WLWREGQR	974.4843	974.5026
SSFAEAAGYCR	1161.4993	1161.5265
DSLHANLQQR	1181.6022	1181.6262
SCYWFSTIK	1205.566	1205.5936
YLESHLAIISNK	1387.7579	1387.7778
IPYTNSLHANLQQR	1654.866	1654.8976
ERIPYTNSLHANLQQR	1940.0096	1940.0448
LGEAFNYWLGASDLNIEGR	2125.0349	2125.0723
YLESHLAIISNKDEDSFIR	2250.1401	2250.1763
MNYTNWSPGQPDNAGGIEHCLELR	2773.2456	2773.2913

Mass fragments of tryptic peptides extracted after SDS-PAGE identified the 46.8 kDa protein as the full length His-tagged GFP-perlucin including the two C-terminal repeat motifs “DSLHANLQQR” and “NSLHANLQQR” (Figure 4E).

**Figure 5:**
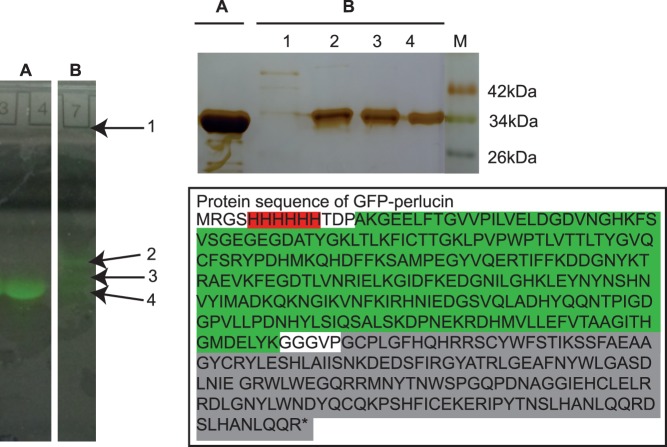
Native PAGE of GFP (28.9 **kDa), GFP-perlucin (46.8** **kDa), and SDS-PAGE analysis of extracted GFP-perlucin derivatives.** Purified GFP yielded one distinct band (A). Affinity-purified GFP-perlucin (B) migrates in 3 separate fluorescent bands (2–4) in native PAGE and another fluorescent species remains in the well (1). The latter contains the full-length GFP-perlucin (46.8 kDa) as demonstrated by denaturing SDS-PAGE under reducing conditions and silver staining (bottom right, amino acid sequence of perlucin (grey) with affinity tag (red) and GFP (green)). Bands 2–4 were identified as truncated versions of the recombinant protein (29.8–30.3 kDa) by MALDI-TOF/TOF (Figure 3). Molecular weight marker (M).

Native polyacrylamide gel electrophoresis revealed only one fluorescent band for GFP (Figure 5A), a 28.9 kDa protein according to mass spectrometry results (Figure 3, top). The 29.8–30.3 kDa GFP-perlucin variants migrated in three separate bands in native PAGE (Figure 5B), whereas the 45–47 kDa GFP-perlucin remained stuck in the well, indicating its tendency to aggregate. The top portion of the gel, presumably containing the full-length GFP-perlucin (46.8 kDa), was analyzed by denaturing SDS-PAGE under reducing conditions. Silver staining of this gel (Figure 5, top right B lane 1) revealed a minor band corresponding to either the 410aa or 395aa GFP-perlucin variants (∼45–47 kDa, Figure 4; aa, amino acid). Bands 2–4 obtained in native PAGE (Figure 5, left B) were identified as truncated versions of the recombinant protein (29.8–30.3 kDa) by MALDI-TOF/TOF (Figure 3) and denaturing SDS-PAGE (Figure 5, top right B, lanes 2–4). For comparison, the full-length sequence of the His-tagged GFP-perlucin protein is shown in Figure 5, bottom right.

**Figure 6:**
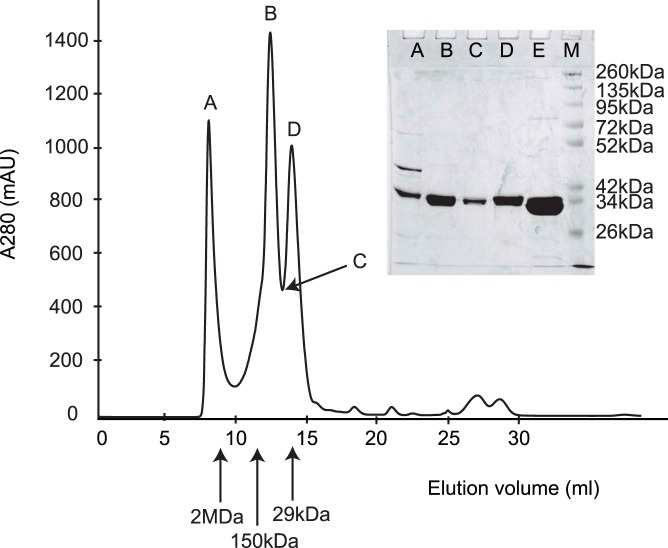
Size exclusion chromatography of GFP-perlucin variants. An elution profile of a typical GFP-perlucin extract was obtained by size exclusion chromatography and analyzed by SDS-PAGE. Soluble protein complexes of the 395aa and 410aa variants of GFP-perlucin (45–46.8 kDa, see Table 1 and Figure 4D,E, compare also Figure 5B, lane 1) and of the truncated variants elute first (peak A) at a volume comparable to 2,000 kDa standard. The truncated variants (29.8 kDa and 30.3 kDa, see Figure 4B,C) elute separately (B–D) at elution volumes comparable to the 150 kDa standard (peak B) and a second peak fraction comparable to the 29 kDa standard (peak D). Note that these two fractions are clearly separated (C). For control (E), soluble GFP (28.9 kDa, Figure 4A) migrated slightly faster on the SDS-polyacrylamide gel than the truncated variants (B–D) of the GFP-perlucin fusion protein. (M) Molecular weight marker. Arrows indicate the size standards: Dextran (V_e_ = 8.9 ml, 2,000 kDa); carbonic anhydrase (V_e_ = 14.12 ml, 29 kDa), ß-amylase (V_e_ = 11.02 ml, 150 kDa).

An additional size exclusion chromatography step was performed in order to separate the full length and truncated protein variants (Figure 6). Although one of the peak fractions (Figure 6, peak A) did contain the full length fusion protein, it co-eluted as a minor fraction together with the truncated variants. The remaining proteins produced a splitted peak in the elution profile (Figure 6B–6D), which migrated in almost confluent bands in SDS-PAGE with relative mobilities corresponding to the truncated protein variants. However, the migration pattern was characteristic for the different protein variants in native PAGE as shown in Figure 5B. This indicates that all variants of distinct size, which at least in the case of the full-length protein were separated only under denaturing conditions (SDS-PAGE), formed soluble multi-protein complexes. One can estimate from the size exclusion chromatography results that the 395aa and 410aa variants (45–47 kDa) eluted at a volume V_e_ = 8.1 ml, which corresponded approximately to dextran standards of ∼2 Mega-Dalton (V_e_ = 9 ml) (Figure 6, peak A). This indicated that soluble protein complexes consisting of about 50 individual polypeptide chains were formed. In the case of smaller complexes (Figure 6, peak B), which eluted at V_e_ = 12.6 ml, the corresponding size standard (V_e_ = 11.7 ml, alcohol dehydrogenase, 150 kDa) indicated an approximate size of <150 kDa. This corresponded well with soluble complexes of about 3–5 individual polypeptide chains. However, in this case no 45 kDa –47 kDa variants were present in these complexes. Peak D (Figure 6) eluted at V_e_ = 14.2 ml and thus likely represented the soluble monomer of the smallest truncated variants (29.8 kDa–30.3 kDa). The size standard (carbonic anhydrase) eluted at V_e_ = 14.12 ml (29 kDa). The two peaks B and D are clearly separated (Figure 6C), indicating that the transition from soluble complexes of 3–5 units to the monomer is discontinuous. The His-tagged GFP (Figure 6E) migrates slightly faster on the SDS-PAGE than proteins from the size exclusion eluates (Figure 6A–D).

### Function of Perlucin-tagged GFPs in Calcium Carbonate Systems

**Figure 7:**
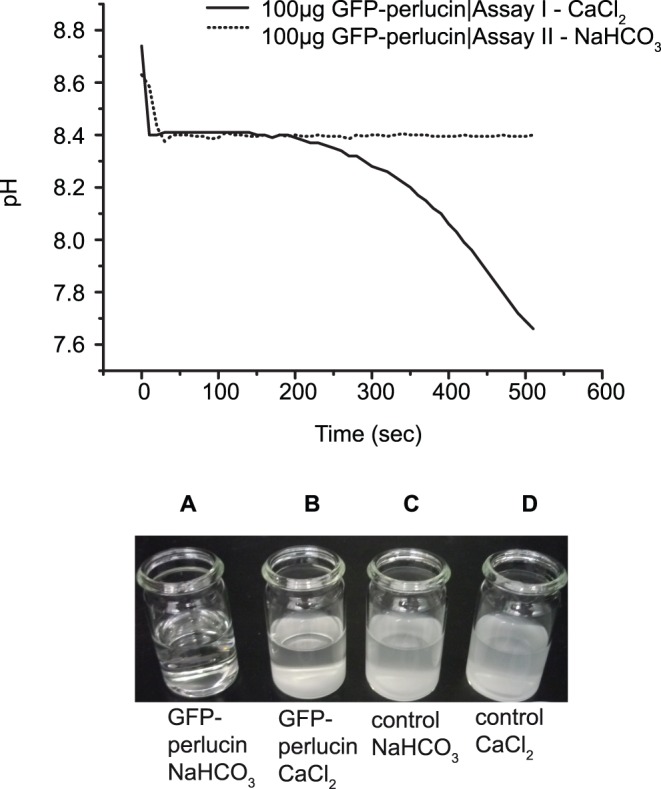
CaCO_3_ precipitation assays I and II. The inhibitory effect of high protein concentrations on the precipitation of calcium carbonate depends on the initial protein precursor solution. The presence of 25–100 µg GFP or GFP-perlucin in a NaHCO_3_ precursor solution delays the precipitation of calcium carbonate when Ca^2+^ is added to this solution (top, dotted line; bottom, vial A). A protein/CaCl_2_ precursor solution precipitates within ∼10 min from the addition of HCO_3_^–^ as indicated by the pH curve (top, solid line) and yields a white precipitate (bottom, vial B). While the time-course of the pH drop observed in control experiments without protein additives is similar to the protein/CaCl_2_ precursor, regardless of which one of the solutions is present in the vial and which one is added, the turbidity of the suspensions (bottom, vials C and D) differ from protein/CaCl_2_ precursors (bottom, vial B). The changes of pH values during the initial time intervals of precipitation are summarized in Table 2.

The influence of one-step purified GFP and GFP-perlucin derivatives on the precipitation of calcium carbonates were assessed in two different supersaturation assays (Figure 7). The assays (assay I and II) differed in the precursor solution to which the protein eluate was added first (protein-Ca^2+^ and protein-HCO_3_^–^ -precursor).

In the assay I with protein-Ca^2+^ precursor, one-step purified, dialyzed GFP-perlucin (5 µg, 25 µg, 100 µg), GFP (100 µg), and Concanavalin A, used as a lectin control protein (25 µg, 100 µg), were diluted in 6 ml 20 mM CaCl_2_. When each solution was poured into equal volumes of NaHCO_3_ solution at pH 8.70±0.11, the pH decreased faster with 5 µg protein (0.4 µg/ml) than with 100 µg (8 µg/ml) to a final pH of 7.5±0.1 (the reaction was completed after 600 seconds as observed in all experiments). After 400 seconds (Table 2), the pH was 7.6±0.2. All calcium carbonate suspensions contained the same small-grained, white precipitates (Figure 7, vial B and Figure S2).

**Table 2:** Calcium carbonate precipitation: Effect of protein additives and concentrations.

Sample	n	pH 0 sec	ΔpH 0–20 sec	pH 20 sec	ΔpH 20–400 sec	pH 400 sec
5 µg GFP-perlucin|CaCl_2_	3	8.84+/−0.03	−0.3	8.54+/−0.01	−0.95	7.59+/−0.03
25 µg GFP-perlucin|CaCl_2_	5	8.82+/−0.09	−0.36	8.46+/−0.05	−0.7	7.76+/−0.2
100 µg GFP-perlucin|CaCl_2_	1	8.74	−0.34	8.4	−0.34	8.06
100 µg GFP|CaCl_2_	4	8.52+/−0.04	−0.26	8.26+/−0.03	−0.29	7.97+/−0.22
100 µg Concanavalin|CaCl_2_	1	8.81	−0.4	8.41	−0.98	7.43
25 µg Concanavalin|CaCl_2_	3	8.70+/−0.02	−0.37	8.33+/−0.01	−0.92	7.41+/−0.01
5 µg GFP-perlucin|NaHCO_3_	4	8.79+/−0.06	−0.3	8.49+/−0.05	−0.98	7.51+/−0.05
25 µg GFP-perlucin|NaHCO_3_	4	8.79+/−0.11	−0.3	8.49+/−0.07	−**0.01**	8.48+/−0.03
100 µg GFP-perlucin|NaHCO_3_	2	8.63+/−0.03	−0.2	8.43+/−0.01	−**0.03**	8.40+/−0.01
100 µg GFP|NaHCO_3_	4	8.52+/−0.05	−0.22	8.30+/−0.03	−**0.01**	8.29+/−0.02
100 µg Concanavalin|NaHCO_3_	1	8.76	−0.32	8.44	−0.97	7.47
25µ g Concanavalin|NaHCO_3_	3	8.66+/−0.02	−0.3	8.36+/−0.02	−0.93	7.43+/−0.02

The effect on pH drop is illustrated by selected time points at t = 0 sec, 20 sec and 400 sec. The initial ΔpH (0–20 sec) is 0.3±0.1 for all experiments (n = number or replications). Significant differences become visible only after several minutes. For HCO_3_^–^ as first ionic interaction partner, the second pH shift (ΔpH 20–400 sec) is lacking at high GFP and GFP-perlucin protein concentrations (25 µg and 100 µg) (values highlighted in bold). For Ca^2+^ as the first ionic interaction partner, the second pH shift (ΔpH 20–400 sec) is reduced compared to experiments with low protein concentration (0.4 µg/ml), or with Concanavalin A (2 µg/ml or 8 µg/ml). This demonstrates that the assay is extremely sensitive to certain parameters such as protein concentration and selected precursor solution in which the protein is supplied first.

**Figure 8:**
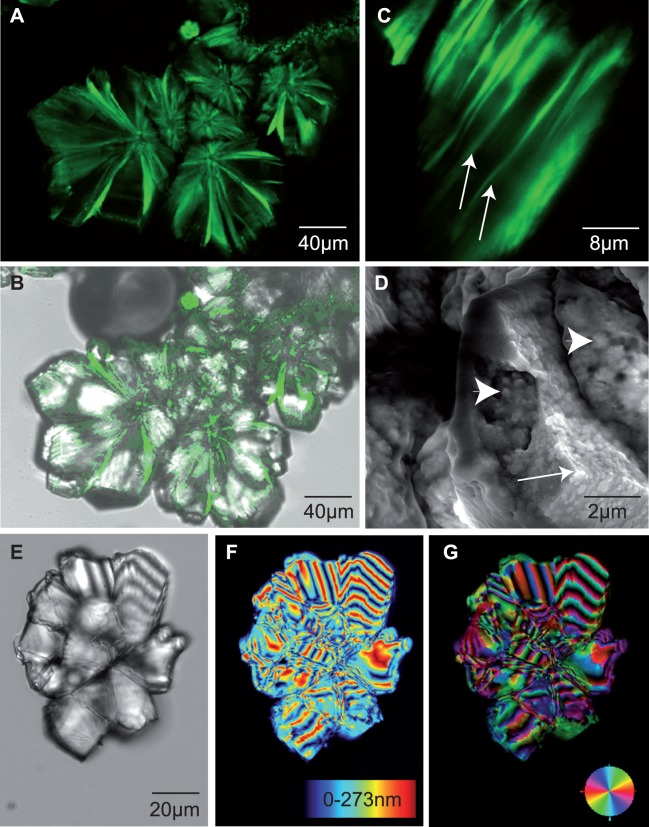
Calcium carbonate morphology in assay II (protein/NaHCO_3_ precursor solution). Confocal fluorescence laser scanning microscopy images (A–C) demonstrate that GFP-tagged proteins are intimately associated with the mineral phase (B, DIC and fluorescence overlay image) in a layered arrangement of radially expanding (A,B) or lamellar sandwiched (C) aggregates. The mineral platelets of the specimen shown in (C) are several µm thick and have an internal sub-micron structure as revealed by scanning electron microscopy (D). The platelets are separated from each other by thin, homogeneous layers (arrows) of organic material which correspond with the fluorescence signals in (C). An LC-PolScope analysis ((E–G); (E), bright field; (F), birefringent retardance; (G), orientation of slow axis vector) of mineral precipitates obtained in the presence of GFP-tagged proteins reveals a characteristic spherulitic structure. Note that this was the predominant form among variable crystal morphologies obtained in presence of protein.

In the Assay II with protein-HCO_3_^–^ precursor, the protein concentration played a major role. At low protein concentrations (5 µg, 0.4 µg/ml) the pH dropped within the first 400 seconds was similar to assay I. Surprisingly, at protein concentrations of >2 µg/ml, pH values remained stable for more than 400 seconds. No visible precipitates were obtained with GFP-perlucin, or GFP (100 µg, 8 µg/ml), once the initial pH shifted from 8.70±0.11 to 8.40±0.07 within 20 seconds after combining the two solutions (Table 2). Within 2–72 hours, crystals formed free-floating and strongly attached to the bottom of the glass vial from a clear, transparent solution (Figure 7A and Figure 8)_._ In contrast to GFP and GFP-perlucin, the same concentrations of FITC-labelled Concanavalin A, a lectin control protein, did not delay or inhibit calcium carbonate precipitation (see Table 2 for pH values).

We investigated further the morphologies of calcium carbonate precipitates obtained in both assays with one-step purified recombinant His-tagged GFP-perlucin, His-tagged GFP, and commercial FITC-labelled Concanavalin A. Small-grained white precipitates, which formed within the first 20 seconds in the assay I, were either spherical or rhombohedral (Figure S2A). Fluorescence spots with a broad range of sizes were observed within the majority of crystals, some of them with weakly fluorescent edges (Figure S2B). Most of the precipitates appeared polycrystalline according to LC-PolScope birefringence analyses (Figure S2C,D). Some fluorescent precipitates appeared not or only slightly birefringent. Increased fluorescence was usually associated with spherical structures. Disordered agglomerates were favoured, indicating a rather non-specific interaction and uncontrolled co-precipitation of proteins and mineral phases.

Some crystals of several tens of microns in size appeared after 3 days from initially inhibited precipitation assays (assay II, 25–100 µg GFP-perlucin (2–8 µg/ml) and 100 µg GFP (8 µg/ml) in HCO_3_^–^ precursor solutions; Figure 7, vial A). These frequently yielded characteristic morphologies of a frost pattern type (Figure 8A,B). Confocal laser scanning fluorescence microscopy of the intact mineral precipitates (Figure 8C) and scanning electron microscopic analyses (Figure 8D) revealed an internal lamellar structure of the composite crystals. Fluorescence signals accumulated preferably at the interfaces between the lamellae (Figure 8C, arrows). Single lamellae were about 4–5 µm thick and consisted internally of submicron spherical building blocks (Figure 8D, arrowheads). The edges of the mineral lamellae were sharp (Figure 8D, arrow). The lamellar arrangement and changes in thickness of the platelets produced characteristic patterns in the LC-PolScope birefringence analysis (Figure 8E,F,G).

## Discussion

What puts a limit to understand how proteins involved in biomineralization exert control, are the distinct and complex interaction potentials of these proteins with each other and, on the other hand, with mineral precursors [29]. Protein amino acid composition and specific properties such as net charge [25] and reversible and irreversible agglomerates may have key regulatory functions in crystal growth [30]. Proteins involved in biomineralization may behave differently in bulk and on surfaces, which are dynamic systems in the case of forming biominerals (see for example, [31]). All this makes the extraction and characterization of both, native and biotechnologically produced proteins involved in biomineralization in functional form rather difficult.

With respect to biomineralization, no particular function was known for the green-fluorescent protein (GFP) from the non-mineralizing marine organism *Aequorea victoria* [32] until it was found that GFP incorporates into metastable calcium carbonate mineral phases such as vaterite while maintaining its fluorescence properties [28]. Here, we intensely characterized GFP-tagged recombinant perlucin from the abalone’s mother-of-pearl. This C-type lectin perlucin was previously known to enhance the precipitation of calcium carbonate in solution [13], [18]. We now investigated the effects of the two antagonistic proteins (i.e. inhibition and promotion of mineral precipitation). The insoluble mineral promoting domain became more soluble due to the fusion with the mineral inhibiting domain. This facilitated the biotechnological purification, on the one hand, but on the other it also interfered with mineral formation. Our experiments showed that the natively purified recombinant dual-function proteins yield bio-fluorescent composites with a layered structure of multiple hierarchical levels. Certain conditions favoured the accumulation of the protein at mineral interfaces. The activity of recombinant His-tagged GFP and GFP-perlucin fragments depended, besides pH, also on the primary ionic interaction partner. The latter was in this case either Ca^2+^ cations or HCO_3_^–^ anions (compare also [33], [34]). In contrast, the incorporation of Concanavalin A-FITC in bulk assays was never related to inhibitory effects or delayed mineral precipitation.

It is known from protein crystallography studies and analytical ultracentrifugation that GFP usually forms monomers or weakly associated (K_D_ ∼100 µM) dimers [35], [36]. At present, it is unknown how exactly the GFP molecules interact with each other in the lamellar arrangements of Fig. 8C of our present study and whether or not they form crystal-like arrangements as for example shown for GFP-ubiquitin fusion proteins in Fig. 1b of [37]. According to recent studies it seems unlikely that dimerization or agglomeration would alter the spectroscopic properties [36], [38]. Suitable linkers, cyclization and affinity tags could certainly be useful in order to avoid expression problems ([39], [40], [41], [42], [43], [44], [45], [46], [47] and Refs. therein) and to fine-tune GFP dimerization and crystallization [39], [48], [49], [50].

In general, GFP fusion proteins have so far been crystallized under conditions of high protein concentrations (e.g. 10–40 mg/ml [37], [39], [51]), whereas in the present study protein concentrations were in the range of µg/ml and precipitation occurred within 3 days. Protein crystallization usually requires high ionic strength, and is performed under conditions which favour slow crystal growth [51]. While engineered fluorescent proteins are frequently used to detect intracellular calcium in solution [52], the influence of divalent cations on the crystallization of GFP variants has not yet been systematically characterized (e.g. 50 mM Cd^2+^, [37]). Ca^2+^ binding sites of GFP [53], [54], [55] may vary in their affinity for divalent cations in solution and in the presence of biointerfaces [48], [56], [57].

GFP thus offers the opportunity to design various biomineralization fusion proteins and to study their interactions with biosynthetic mineral phases. In particular, the inhibitory effect of GFP bears not only the potential to control biomineral morphology. Since GFP incorporates into calcium carbonate minerals and perhaps even maintains its fluorescent properties, the distribution of protein constituents can be monitored within transparent mineral phases using light microscopy. The principle of creating fusion proteins with dual function – inhibitory and promoting with respect to mineral phases, as well as the formation of defined protein aggregates – may be applied in the future for producing biomimetic crystals.

## Materials and Methods

### Molecular Cloning

Recombinant GFP-perlucin was obtained using pQE expression systems (Qiagen, Hilden, Germany) based on the previously described pQE31-GFP-CBP vector [58]. The sequence encoding perlucin (UniProtKB accession no. P82596) was achieved by reverse translation. The synthetic gene (Entelechon, Regensburg, Germany) was subcloned into pENTR/D-TOPO (Invitrogen, Darmstadt, Germany), which served as the template DNA. *Kpn*I (5-Perlucin_KpnI_pQE31 primer sequence 5′ CGGGGTACCCGGATGTCCTTTGGG) and *Pst*I (3-Perlucin_PstI primer sequence 5′ TGCACTGCAGTTATCTTTGTTGCAGATTGG) restriction sites were introduced by PCR cloning and ligation into *Kpn*I and *Pst*I digested pQE31-GFP-CBP vector [58]. Selected pQE31-GFP-perlucin clones with the correct sequence (Eurofins MWG, Ebersberg, Germany) were transformed in *E. coli* XL-1Blue (Stratagene, Amsterdam, the Netherlands). The control plasmid used for reference experiments containing only the GFP sequence was derived from the pQE31-GFP-CBP vector [58] by deletion of the C-terminal CBD sequence.

### Protein Expression and Purification

Recombinant GFP-perlucin protein was expressed in XL-1Blue *E. coli* cells as described by [15] with major modifications. Cells from single colonies on agar plates were cultivated for 14 hours in 2.5 ml Luria broth (LB) medium at 37°C, 180 rpm for inoculation of 50 ml LB medium. Cells were grown to O.D.600 ∼0.5–0.7 prior to 0.5 mM IPTG induction (Fermentas, St. Leon-Rot, Germany) and continued to grow at 22°C for 8 hours and at 18°C for 16 hours at 180 rpm. Cells were harvested by centrifugation at 3,200×g for 30 minutes at 4°C according to the manufacturers instructions [59], and shock frozen in liquid nitrogen. After thawing on ice, cells were resuspended in 2 ml lysis buffer for purification under native conditions per 0.5 g wet weight [59], supplemented with 1 mg/ml lysozyme (Roth, Karlsruhe, Germany) and incubated on ice for 30 minutes. After centrifugation at 16,000×g, 4°C for 45 minutes in a JA25.50 rotor (Beckmann, Krefeld, Germany), the supernatant fraction was collected. This crude extract was 0.22 µm filtrated and subjected to affinity purification using 50 µl (50%) Ni^2+^-NTA matrix (Ni^2+^-NTA Agarose, Qiagen, Hilden, Germany) per 50 ml culture. His-tagged proteins were bound in batch to the matrix for 1 h at 4°C under rotation. Washing steps and elution was performed according to the manufacturer’s instructions for native purification [59] using columns with 10 µm pore size filters (M2110, Mobitec, Göttingen, Germany). The GFP used for control purposes was purified from the respective host cells grown, harvested and natively extracted according to standard procedures as described in [59]. The purified GFP-perlucin was concentrated in centrifugal membrane devices (3 kDa MWCO, Pall, Ann Arbor, USA). The concentrated protein solution (∼20 mg/ml) was subjected to size exclusion chromatography using an ÄKTA Purifier system equipped with a Superose12 10/300 GL column (GE Healthcare, Munich, Germany). Chromatography was performed in lysis buffer [59] at a constant flow rate of 0.4 ml/min and a detection wavelength of 280 nm. Peak fractions were subsequently analyzed. Gel filtration molecular weight markers in the size range of 12 kDa –2 MDa (Mega-Dalton) were obtained from Sigma-Aldrich (# MWGF200, Sigma-Aldrich, Munich, Germany).

### Protein Analysis

Standard SDS-PAGE (sodium dodecyl sulphate polyacrylamide gel electrophoresis) was performed under reducing conditions. Protein bands were stained according to [60]. A Spectra™ Multicolor broad range ladder (#SM1841, Fermentas, St. Leon-Rot, Germany) was used as a molecular weight (M.W.) marker.

Native PAGE was performed using Mini Protean TGX Gels (10%) (Biorad, Munich, Germany). Protein samples were recovered from native page through diffusion from equal gel volumes in 10 mM Tris pH 8.7 for 24 hours. Prior to activity assays, native protein fractions were analyzed after extraction by SDS-PAGE under reducing conditions for comparison. Detection of fluorescence fusion proteins immediately after separation in the native page were performed with Fluorchem Q bioillumination system (Biozym, Hessisch Oldendorf, Germany) and FluorchemQ software package.

Molecular weight analysis was performed with matrix assisted laser desorption/ionisation mass spectroscopy (MALDI-TOF/TOF). The intact protein mass of native GFP and GFP-perlucin were measured directly from the chromatography eluates. In order to identify GFP and GFP-perlucins, mass fragments were analyzed. Samples were prepared on a 10% SDS Gel. The protein bands of interest in the SDS gel were cut off and subjected to in-gel tryptic digestion with 0.2 µg/µl trypsin (sequencing grade, Roche, Mannheim, Germany) according to established protocols [61].

### CaCO_3_ Precipitation Assays

Two different calcium carbonate precipitation assays were developed based on previously established procedures with major modifications [13], [23]. Protein samples (GFP, GFP-perlucin derivatives), and control proteins FITC-Concanavalin A (#C7642, Sigma-Aldrich, Munich, Germany) were dialyzed (MWCO 4,000–6,000 Da, Roth, Karlsruhe, Germany) against 10 mM Tris buffer pH 8.7 immediately prior to each assay to avoid early salt induced precipitation by protein purification buffers. Chemicals were A.C.S. grade and obtained from Merck or Roth, if not stated otherwise. All solutions were prepared with deionized water (MilliQ, Millipore, Schwalbach, Germany) and 0.22 µm filtered. All glass equipment was thoroughly rinsed with deionised water immediately before use. All precipitation assays were accomplished at room temperature 22°C ±2°C.

#### Assay I-CaCl_2_

A volume of 6 ml 20 mM CaCl_2_ solution (3 mM Tris pH 8.7) was supplemented without or with protein solutions of (a) GFP-perlucin: 5 µg, 25 µg, or 100 µg, b) GFP: 100 µg, c) Concanavalin A: 25 µg or 100 µg), 0.22 µm filtrated and poured into 6 ml 20 mM NaHCO_3_ (3 mM Tris pH 8.7) at constant rate on a Teflon-coated magnetic stirrer. The final protein concentrations in 12 ml were 0.4 µg/ml (5 µg), 2 µg/ml (25 µg) and 8 µg/ml (100 µg).

#### Assay II-NaHCO_3_

A volume of 6 ml 20 mM NaHCO_3_ solution (3 mM Tris pH 8.7) was supplemented without or with protein solutions of (a) GFP-perlucin 5 µg, 25 µg, or 100 µg, b) GFP: 100 µg, c) Concanavalin A: 25 µg or 100 µg), The solution was 0.22 µm filtrated and poured into 6 ml 20 mM CaCl_2_ (3 mM Tris pH 8.7) at constant rate on a Teflon-coated magnetic stirrer. The final protein concentrations in 12 ml were 0.4 µg/ml (5 µg), 2 µg/ml (25 µg) and 8 µg/ml (100 µg).

The pH values were recorded every 10 seconds for at least 10 minutes from combining the solutions. A Prolab 3000 pH meter (SI Analytics, Jena, Germany) equipped with a N6000 electrode (SI Analytics, Jena, Germany) and MultiLab Pilot V5.04 software (WTW, Nova Analytics company, Germany) was used for automated recording of pH values.

### Fluorescence Microscopy

Fluorescence images were taken using the same Zeiss Observer Z1 equipped with an A Plan 10×/0.25 Ph1, LD Plan Neofluar 20×/0.4Korr Ph2 and a LD Plan Neofluar 344, 40×/0.6Korr Ph2 objectives and with an epifluorescence Colibri illumination system combined with filter set 38HE (Zeiss, Göttingen, Germany). Images were recorded with an AxioCamMRm (60N-C2/3′ ´ 0.63×) digital camera and analyzed using the Zeiss AxioVision software (Zeiss, Göttingen, Germany). The exposure time was automatically adjusted using pre-defined algorithms.

### LC–PolScope Microscopy

All samples were prepared in water on glass slides, if not stated otherwise. An inverted light microscope Zeiss Observer Z1 equipped with an A Plan 10×/0.25 Ph1, LD Plan Neofluar 20×/0.4Korr Ph2 and a LD Plan Neofluar 344, 40×/0.6Korr Ph2 objectives and LC-PolScope technology (CRI Abrio Imaging System, L.O.T. Oriel, Darmstadt, Germany), including the respective filter sets was used to analyze precipitates with respect to birefringent retardance and orientation.

### Confocal Laser Scanning Microscopy (CLSM)

The distribution of fluorescence signals within precipitates was investigated using a TSC SP2 confocal laser scanning microscope (Leica Microsystems CMS GmbH, Mannheim, Germany) equipped with a HCX PL APO 40×/1.25–0.75 oil objective. Samples were mounted wet on glass slides. A 488 nm argon laser was used for GFP excitation and emission signals were recorded between 507–512 nm.

### Scanning Electron Microscopy

An environmental scanning electron microscope (E-SEM Quanta 400, FEI, Eindhoven, the Netherlands) was used for structural surface characterization of precipitates. Sample droplets were directly mounted on a silicon wafer without additional coatings. Images were obtained in Low Vacuum mode at 100Pa pressure at 10–20kV.

## Supporting Information

Figure S1 **Expression and native purification of GFP (28.9** **kDa), GFP-perlucin (46.8** **kDa) and perlucin (20.2** **kDa).** Cell cultures were analyzed using SDS-PAGE analysis in 1-hour intervals (t0–t4) and on the following day (t5–t6). The binding (B), washing (W), and elution steps (E1 and E2) demonstrate the success in native affinity purification of GFP-perlucin. While GFP is easily obtained under standard growth conditions, the yield of natively purified GFP-perlucin depends largely on the culture conditions (top row). Perlucin (20.2 kDa) is expressed, but cannot be recovered in native form, irrespective of the growth conditions (bottom row). Although the GFP domain seems to have a positive effect on the solubility of the recombinant GFP-perlucin (46.8 kDa), additional processing occurs during cell growth as demonstrated by the occurrence of a 30 kDa fragment (34 kDa band, compare also Figure 3, left arrow), which lacks approximately 145 C-terminal amino acids. Identical molecular weight markers (M) were used for all gels (10% acrylamide).
(EPS)

Figure S2 **Calcium carbonate morphology in assay I (protein/CaCl_2_ precursor solution).** Microscopic analyses of precipitates were performed using bright field (A), fluorescence (B) and LC-PolScope in birefringent retardance (C) and orientation of the slow axis vector (D) imaging modes. The comparative analysis of the micrographs shows that fluorescent GFP-perlucin is closely associated and likely incorporates into calcium carbonate crystals. Fluorescence signals appear inhomogeneous in size and distribution in the centre of mineral particles and slightly weaker on the crystal edges.
(EPS)
